# A diagnostic test accuracy study investigating GP clinical impression and brief cognitive assessments for dementia in primary care, compared to specialised assessment

**DOI:** 10.3233/JAD-230320

**Published:** 2023-01-01

**Authors:** Samuel Thomas Creavin, Mark Fish, Michael Lawton, Sarah Cullum, Antony Bayer, Sarah Purdy, Yoav Ben-Shlomo

**Affiliations:** 1 Population Health Sciences, University of Bristol, Canynge Hall, 39 Whatley Road, Bristol BS8 2PS; 2 Royal Devon and Exeter Hospital; 3 Faculty of Medical and Health Sciences, The University of Auckland, Building 507, 22-30 Park Avenue, Grafton, Auckland 1142, New Zealand; 4 Division of Population Medicine, Cardiff University, Cardiff, CF64 2XX

**Keywords:** Alzheimer’s disease, dementia, General Practice, Sensitivity and Specificity, Symptom Assessment

## Abstract

**Background:**

Many health systems are interested in increasing the number of uncomplicated and typical dementia diagnoses that are made in primary care, but the comparative accuracy of tests is unknown.

**Objective:**

*Calculate diagnostic accuracy of brief cognitive tests in primary care*

**Design:**

We did a diagnostic test accuracy study in general practice, in people over 70 years who had consulted their GP with cognitive symptoms but had no prior diagnosis of dementia. The reference standard was specialist assessment, adjudicated for difficult cases, according to ICD-10. We assessed 16 index tests at a research clinic, and additionally analysed referring GPs clinical judgement.

**Results:**

240 participants had a median age of 80 years, of whom 126 were men and 132 had dementia. Sensitivity of individual tests at the recommended thresholds ranged from 56% for GP judgement (specificity 89%) to 100% for MoCA (specificity 16%). Specificity of individual tests ranged from 4% for Sniffin’ sticks (sensitivity 100%) to 91% for TUG (sensitivity 23%). The 95% centile of test duration in people with dementia ranged from 3 minutes for 6CIT and TAC, to 16 minutes for MoCA. Combining tests with GP judgement increased test specificity and decreased sensitivity: e.g., MoCA with GP Judgement had specificity 87% and sensitivity 55%.

**Conclusions:**

Using GP judgement to inform selection of tests was an efficient strategy. Using IQCODE in people who GPs judge as having dementia and 6CIT in people who GPs judge as having no dementia, would be a time-efficient and accurate diagnostic assessment.

## Background

Enhancing the role of general practitioners in making a diagnosis of dementia in uncomplicated cases is a priority in the UK [1], but a barrier for General Practitioners (GPs) is choosing between the wide variety of brief cognitive assessments (BCAs) that are available [2,3]. There is little evidence to help GPs choose between tests; indeed, national guidelines recommend different tests [4].

GPs use time as a diagnostic test [5], and dementia is a progressive disorder which can be difficult to diagnose early in the disease process. However, some people wait a long time for a diagnosis after presenting with symptoms largely due to practical and logistical difficulties in accessing specialist expertise [6]. GPs making a formal diagnosis of dementia in uncomplicated cases can reduce anxiety, avoid unnecessary waiting and investigations for alternative diagnosis [7], and is often needed to access additional social care and support (albeit this being an aspect of system design rather than necessity). Most people who are consulting about memory problems would want to know if they had dementia [8]. There may also be benefits to explicitly recognising more advanced dementia in people who have lost capacity and insight, as this can prompt a holistic re-evaluation of care-goals and avoid unnecessary tests and treatments [9]. There are currently no widely available disease modifying treatments for dementia, and a focus on highly sensitive tests that does not “miss” any cases of dementia in primary care may result in an overwhelmed secondary care service with unclear benefits for individual patients and families [10].

The available evidence typically has limited applicability for GPs evaluating people with possible dementia in primary care. Firstly, current studies often report diagnostic accuracy in an asymptomatic population and therefore are more applicable to screening or case finding [11–13] than helping to support a diagnosis in those presenting with clinical symptoms, who have higher pre-test probability of disease. Secondly, studies often investigate accuracy for the target condition of all cognitive impairment [14], rather than specifically dementia, typically with the implicit assumption that all people with cognitive impairment including both those with dementia and those with mild cognitive impairment (MCI) require specialist referral. Thirdly, studies typically investigate a single test in isolation, rather than making direct comparisons between tests in the same study [15], allowing only indirect comparisons. Fourthly, studies often investigate the accuracy of tests in (high prevalence) specialist settings rather than in the (relatively low prevalence) general practice population, and test accuracy is related to prevalence and the spectrum of disease severity [16]. Finally, existing studies tend to evaluate the accuracy of tests alone rather than when combined with GP judgement which is what happens in “real world” clinical practice.

To address the limited applicability of existing studies to primary care, we conducted a study in people with symptoms of possible dementia who had consulted about these with their GP. The aim of this study was to quantify the test accuracy of a range of non-specialist candidate tests, suitable for use in a GP clinic for the evaluation of cognitive symptoms, alone and in combination with GP judgement, compared to a reference standard specialist assessment according to ICD-10 criteria.

## Method

### Population

We recruited participants consecutively from 21 participating GP clinics from a total 82 practices in the Bristol, North Somerset, and South Gloucestershire (BNSSG) area between March 2015 and May 2017. Research clinics took place in four participating GP clinics. A minimum sample size of 200 was needed, based on a specificity of 95% in prior studies and a 75% prevalence of dementia in local memory clinic data [17].

Participants were people with cognitive symptoms but no prior diagnosis of dementia, who were aged at least 70 years and had been referred by their GP to this research study. Cognitive symptoms were not specified but generally include disturbance in memory, language, executive function, behaviour, or visuospatial skills [18]. Symptoms were required to be present for at least six months, and could be reported by the person themselves, a family member, a professional, or another person; there was no severity threshold. Symptom duration was determined from the clinical history. Cognitive symptoms did not need to be the main focus of the consultation with the GP: an enquiry about cognition could be initiated if there was a perception of a problem.

People were excluded if they had a known neurological disorder (i.e. Parkinson’s disease, Multiple Sclerosis, learning disability, Huntington’s disease), were registered blind, or profoundly deaf (i.e. unable to use a telephone), had a psychiatric disorder requiring current secondary care input, or if cognitive symptoms were either rapidly progressive or co-incident with neurological disturbance. People with cognitive problems that were so advanced that they were unable to consent were excluded.

GPs were encouraged to refer a consecutive series of eligible patients with cognitive symptoms. The research team took written consent from all participants. An accompanying informant was mandatory and informants also gave written informed consent to participate. All participants were offered free accessible transport and translation services. All methods were carried out in accordance with relevant guidelines and regulations including Declaration of Helsinki. The National Research Ethics Service Committee London – Bromley (reference 14/LO/2025) gave a favourable ethical opinion on 25 November 2014. NHS Research and Development approvals were granted by Avon Primary Care Research Collaboration on behalf of Bristol, North Somerset and South Gloucestershire clinical commissioning groups. The University of Bristol acted as Sponsor.

### GP Judgement

The referring GP recorded their clinical judgement using an electronic referral form during a consultation with their patient. GP judgement was operationalised as normal, cognitive impairment not dementia (CIND), or dementia as options for response to the question “Is your gut feeling that this person has…”. GPs were not specially trained, were not required to arrange any test, and could refer people simultaneously or subsequently to NHS services. We specifically instructed GPs that they did not need to use any prior cognitive test (we did not mention the index tests by name as we judged on balance this could increase the risk they may be used). The study team contacted the practice at least three times to obtain any missing referral data.

### Index tests

The index test battery was selected following a review of the literature and on the basis of the following criteria: not copyright and either previously evaluated in primary care or not previously evaluated in primary care but of interest [19]. The index assessment included eight brief cognitive assessments, three physical tests, and five informant evaluations. Index tests were all performed by the same examiner (STC), a medical doctor undertaking postgraduate training in general practice and a PhD in diagnostic tests. We conducted index tests as instructed by the original authors and followed instructions of the original test authors for clock scoring. We used prespecified test thresholds which are provided in Supplementary Table 1. We calculated time taken for each test in minutes from the start to end time of each test. The eight brief cognitive assessments were the memory alteration test, M@T [11]; Eurotest [20]; Phototest [21]; Scenery Picture Memory Test, SPMT [22]; Six item cognitive impairment test, 6CIT [16]; general practitioner assessment of cognition, GPCOG [23]; Mini-cog [24]; Time and Change, T&C [25]. The three physical tests were Timed Up and Go (TUG) [26,27]; extra-pyramidal signs scale, EPSS [28]; and Sniffin’ sticks [29]. The five informant questionnaires were the Pfeffer FAQ [30]; Lawton IADL [31]; Katz ADL [32]; 8-item AD8 [33]; and short form IQCODE [34]. Where possible, items were not repeated, e.g. the 6CIT [16] and the GPCOG [23] both require the recall of a 5-item name and address, and to avoid burdening and potentially confusing participants this item was done once and then scored separately for each test.

The Montreal Cognitive Assessment, MoCA [14] was initially not included in the index battery as it was originally designed to diagnose or identify MCI, had been advocated for use in secondary care [35] and had not been investigated in primary care [36]. We revised the protocol in light of subsequent policy changes in 2015 that encouraged GPs to diagnose dementia in typical situations without referring to a specialist [7] using the MoCA as the preferred instrument. We replaced the M@T with the MoCA because we judged that including both the MoCA and the M@T would be overly burdensome for participants and have little added value. We imported Sniffin’ sticks on special order and so we added these at a later stage to avoid delaying the start of recruitment while waiting for this single test.

Excluding Sniffin’ Sticks, we randomly assigned the order of the index tests in the battery for each participant to avoid the effect of order influencing test accuracy. The examiner offered each participant the chance to undertake every test in the battery but was responsive to the participants if they appeared to be becoming tired or distressed.

### Reference standard

At the research clinic, a single specialist physician (JH) with more than 20 years’ experience in the field of dementia conducted a standardised assessment, including the Addenbrooke’s Cognitive Examination (ACE) III [37], Brief Assessment Schedule Depression Cards (BASDEC) [38] and the informant-completed Bristol Activities of Daily Living (BADL) Questionnaire [39], lasting approximately 60 minutes. The specialist was not aware of other test results such as clinical judgement of the referring GP, research clinic index tests, or any investigations. We randomly allocated participants to see the specialist before or after the index tests. All participants were offered, and encouraged, to have a gentle break of 10-20 minutes for a drink and snack between sessions. A second specialist, who had access to the primary care medical record for six months after the research clinic follow-up, as well as all information available to the primary specialist, adjudicated borderline cases. The reference standard was an integrated expert assessment according to ICD-10 criteria [40] for each individual patient, and specific test thresholds were not used; people with CIND were included in the “normal” group for evaluation of test accuracy since we were specifically interested in the test accuracy for dementia. We used the term CIND in the reference standard, while also classifying MCI, for consistency with GP judgement who classified against CIND (GPs being generally unfamiliar with MCI criteria). Study data were electronically entered and managed using REDCap (Research Electronic Data Capture) hosted at the University of Bristol [41].

### Statistical methods

We calculated a potentially eligible population by indirectly standardising the population at risk of dementia in recruiting practices based on age specific incidence of dementia [42] and GP list size [43]. We used a regression model with total ACE-III score as the dependent variable and categorical randomly allocated assessment order (index battery first or specialist assessment) to investigate a possible effect of assessment order on test scores. We calculated the median and range ACE-III total scores for people who were classified by GPs as being normal but in fact had dementia, compared to people who were correctly classified as having dementia. We classified test duration by the 95^th^ centile of test duration, the time which clinicians could expect 95% of people to complete the test. We calculated measures of test accuracy (sensitivity, specificity, likelihood ratios, predictive values) together with 95% confidence intervals. To determine the effect of combining tests with GP judgement we calculated the accuracy of each test combined with GP judgement so that the combined test positive was taken as being both GP judgement positive and the other test positive; and the combined test negative was taken as either both or one of judgement/test negative. We also calculated the diagnostic accuracy when using an approach of “stratified sequential testing”, where clinical judgement determines what further test should be done, by calculating the diagnostic accuracy in cross-tabulations that were restricted based on GP Judgement. We evaluated how GP judgement influenced the discrimination of the index tests by calculating the AUROC (area under the ROC curve) stratified by GP judgement.

In an exploratory analysis we used a bootstrapping procedure with 1000 replications to compare the numbers misclassified, either as false positives or false negatives, from different combinations of cognitive tests, using either an unstratified approach (one cognitive test) or a stratified approach depending on GP judgment. Test combinations for the exploratory bootstrap procedure were chosen on the basis of the three tests with the greatest number of true positives and true negatives for each stratum (unstratified by GP Judgement, GP judgement dementia, GP judgement not dementia).

In practice there is a finite amount of resource available to assess people with symptoms of dementia, and longer assessments mean that fewer people can be evaluated. To account for the trade-offs between test duration and accuracy we derived the numbers of people that a full time (37.5 hours a week) NHS Memory clinician could classify in a population of up to 1000 people, using the (simplified but implausible) assumption that all working time was spent administering cognitive assessments. To derive these figures, we used sensitivity and specificity of each index test stratified by GP judgement, together with 95^th^ centile of test duration also stratified by GP judgement (to account for test duration being longer in people who GPs suspected of having dementia). For informant completed tests which can be completed independently of a clinician we used the time for the shortest duration brief cognitive assessment, since a practical implementation would be for an informant to complete their questions while a clinician evaluated the patient.

All analysis was done using Stata Version 15.

## Results

### Participants

**Fig. 1:**
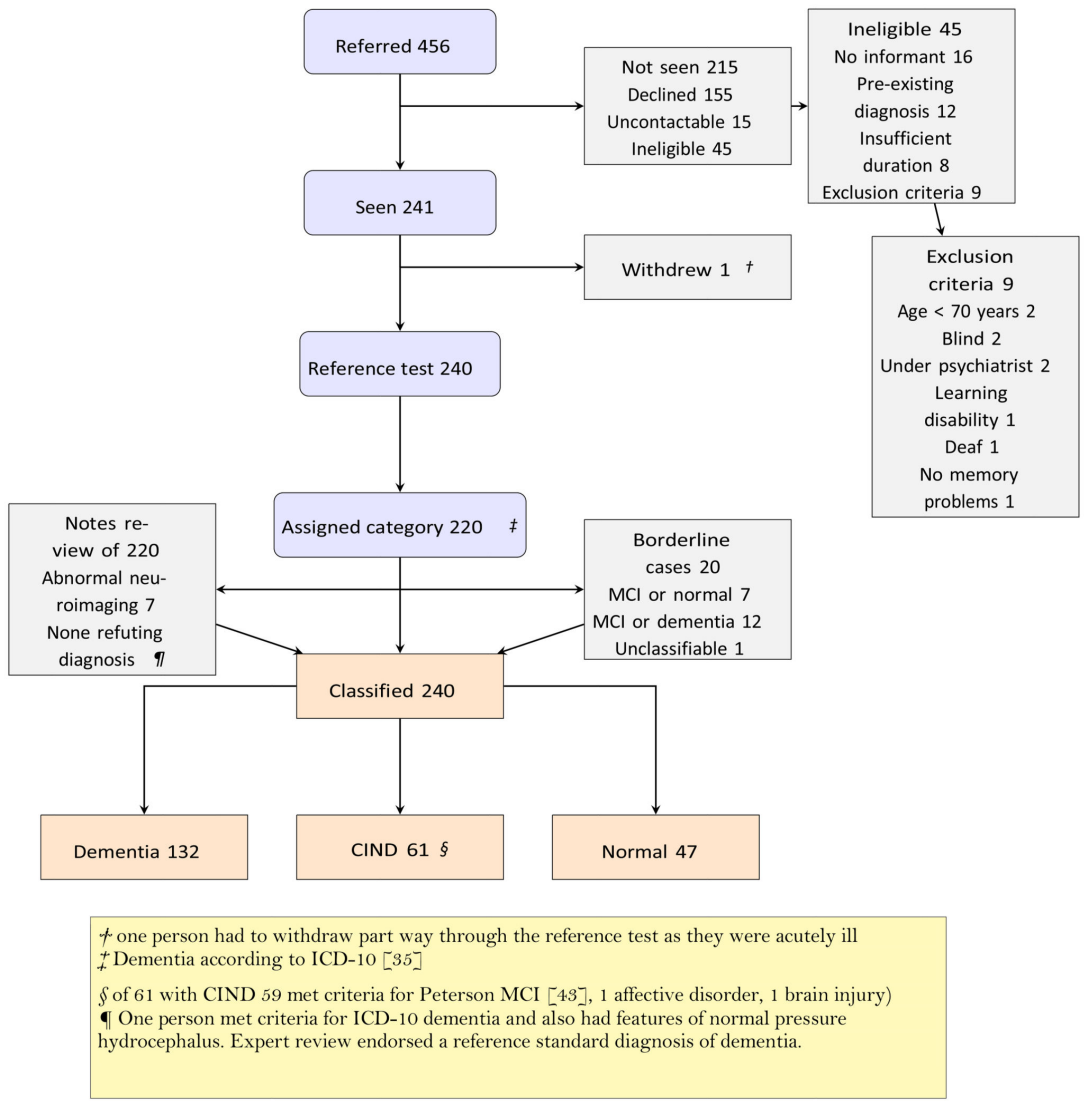
STARDdem flowchart for inclusion of participants in the study

Recruitment is described in detail in a separate paper [19]. Figure 1 shows that GPs referred 456 people, of whom 240 (53%) participated and had available data, 45 were ineligible (10%) and 155 declined (34%). Of 240 participants, 47 were normal, 61 had CIND (59 of whom had Petersen MCI) and 132 had dementia. The median age overall was 80 years (IQR 75 to 85 years), and the median ACE-III total score was 75 (IQR 65 to 87); the median age of leaving education was 15 years (IQR 15 to 16 years) and the median months since symptom onset was 24 months (IQR 12 to 36 months). Using indirectly standardised rates in the recruiting practices we estimate that during the recruitment period around 1,735 people would have been potentially eligible, of whom GPs referred 456 and we saw 241.

GPs judged 34 people as being normal, 120 as having CIND, and 86 as having dementia. People that GPs judged as having dementia had a total ACE-III score IQR of 60 to 74 with a 90th centile of 81/100 and a highest score of 95/100, compared to published ACE-III thresholds of ≤82 for dementia. Six people with dementia were classified as normal by their referring GP, these people had a median ACE-III total score of 72 (range 69 to 82 points) and had a median age of 82 years (range 79 to 86 years). In contrast the 73 people with dementia who were classified as such by their GPs had a median ACE-III total score of 65 (range 26 to 92 points) and had a median age of 82 years (range 71 years to 94 years).

Table 1 presents the characteristics of participants. Of 240 participants, 53% were men, median age was 80 years, median symptom duration was 24 months and the median ACE-III score was 70 out of 100,compared to published ACE-III thresholds of <82 for dementia and < 88 for MCI. Median age of leaving education age was 15 years (range 13 years to 19 years). Table 1 also provides a 3x3 cross tabulation of GP judgement against the reference standard, showing that referring GPs judged that 86 patients (36%) had dementia. In a regression model with total ACE-III score as the dependent variable and test order (index test / reference test first or second) as the independent variable, people who the specialist assessed first scored 2.4 points more on ACE-III (95% CI -1.3 to 6.1) points than those who underwent the index test battery first, after adjusting for age, sex and cognition category they scored 1.9 points more (95% CI -0.8 to 4.5) than those who underwent the index battery first.

### Test characteristics

**Fig. 2:**
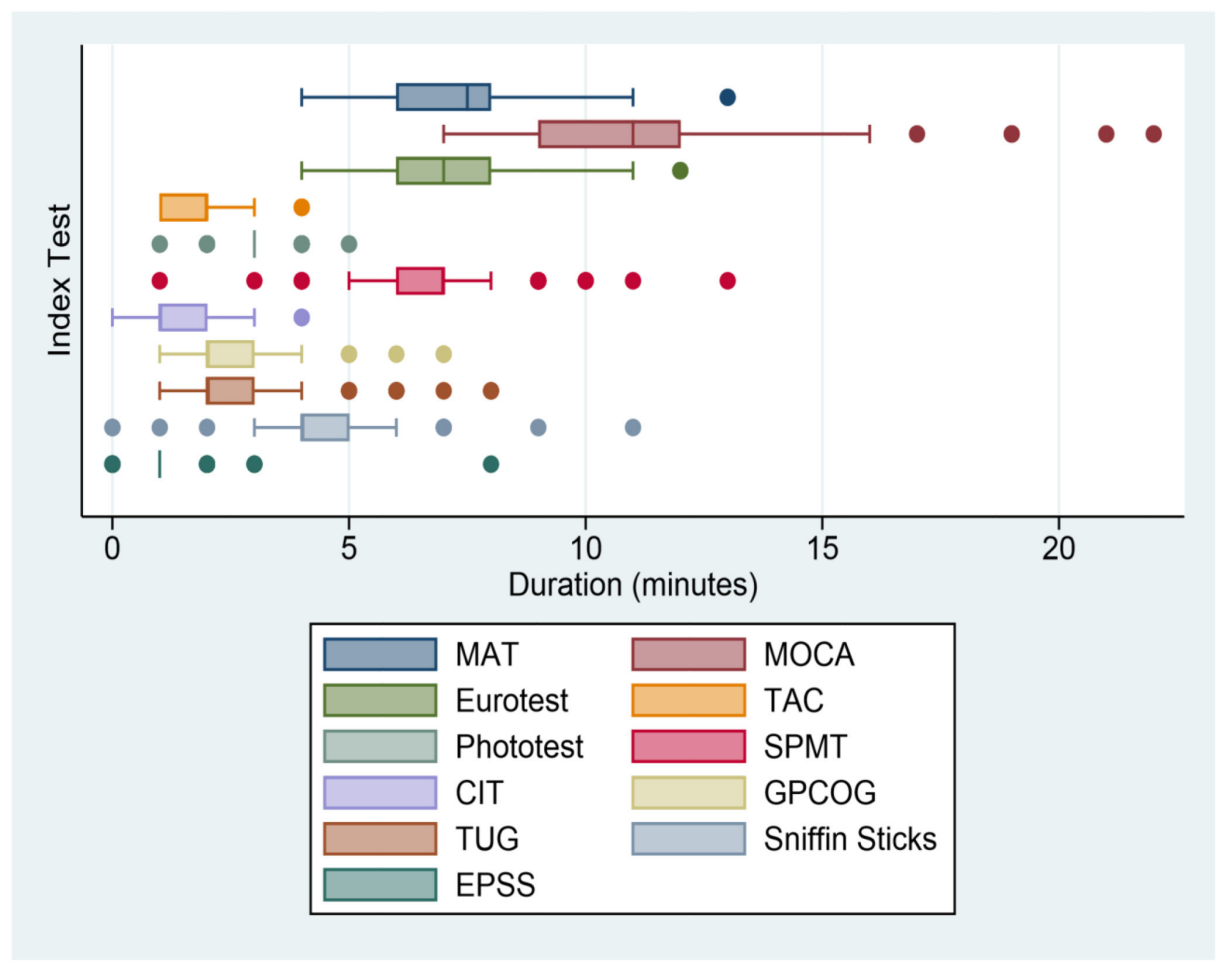
Duration of index tests, all cognition categories *The box plots the median (darker middle line) and the quartiles (box edges), the whiskers enclose the lower (quartile 1 - 1.5 × interquartile range) and upper (quartile 3 + 1.5 × interquartile range) adjacent values, and the dots mark the outlying values*.

There was wide variability in test duration (Figure 2) with the 6CIT having the shortest median duration (1 minute) whereas MoCA had the longest median duration (11 minutes). There was also variation in the range of times taken to complete tests, which differed between tests, for example being much greater for MoCA (range 7 minutes to 22 minutes) than Phototest (range 1 minute to 5 minutes). Classified by the 95^th^ centile of test duration (C95), the short duration (<5 minutes) tests were: EPSS (C95 2 minutes), T&C (C95 3 minutes), 6CIT (C95 3 minutes), Phototest (C95 4 minutes), and GPCOG (C95 4 minutes). The medium duration (≥5 minutes but ≤10 minutes) tests were: TUG (C95 5 minutes), Sniffin’ sticks (C95 6 minutes), SPMT (C95 9 minutes), and Eurotest (C95 10 minutes). The long duration (> 10 minutes) tests were: M@T (C95 11 minutes) and MoCA (C95 15 minutes).

Supplementary Table 1 shows the characteristics of the brief cognitive assessments and physical tests in terms of differential performance by cognitive status and test duration. We observed that performance on every test was worse for people with dementia than people who were cognitively normal, except for the Sniffin’ sticks, and that tests took longer for people with dementia or CIND than people who were normal, though for many tests this could have been due to chance (that is confidence intervals overlapped).

### Diagnostic accuracy

Table 2 indicates that some tests with high sensitivity are better for ruling out dementia whilst others with high specificity are better for ruling in the diagnosis. The tests with the highest sensitivity were MoCA at a threshold of 26 (sensitivity 100%; 95% CI 97% to 100%) and Sniffin’ sticks at a threshold of 11 (sensitivity 100%; 95% CI 96% to 100%). In contrast the tests with the highest specificities were FAQ (specificity 97%; 95% CI 92% to 99%), T&C (specificity 97%; 95% 91% to 99%) and Katz ADL (specificity 95; 95% CI 90% to 98%). GP judgement had modest sensitivity (56%; 95% CI 47% to 65%) but was the third most specific test (89%; 95% CI 81% to 94%).

For many brief cognitive assessments, using tests in combination with GP judgement led to a reduction in sensitivity and increase in specificity. In contrast, informant measures were less affected by combining with GP judgement, although the sensitivity of both AD8 and IQCODE combined with GP judgement were much lower than when these two tests were used alone. When combined with GP judgement the combined test with the highest sensitivity was GP+IQCODE with sensitivity 56% (47% to 64%), and the combined test with the highest specificity was T&C with specificity 100% (97% to 100%).

### Impact of GP Judgement on test performance

Table 3 shows how GP judgement impacts the discrimination of the index tests quantified using the AUROC. SPMT was the test with the highest AUROC overall (AUROC 0.7753 95% CI 0.7220 to 0.8285), and the AUROC was similar regardless of GP judgement for dementia (AUROC 0.7725 95% CI 0.6283 to 0.9168) or not dementia (AUROC 0.7148 95% CI 0.6402 to 0.7894). AUROC was generally higher in people classified as not having dementia than those classified as having dementia. However, the converse was true for TAC, SPMT, M@T, and Eurotest, suggesting that these tests may be more useful in people who GPs judge as having dementia than in people who GPs think do not have dementia.

Table 3 also shows the PPV (positive predictive value) and NPV (negative predictive value) for each test stratified by GP judgement. The predictive values are dependent on GP judgement because this influences the prevalence of disease. PPVs were higher when tests were restricted to GP dementia +, and NPVs were higher when restricted GP dementia -, probably attributable to prevalence effects. PPV for M@T was 100% (95% CI 75% to 100%) in people classified by GPs as having dementia, but only 50% (95% CI 7% to 93%) in people classified as not dementia. In contrast NPV for a normal Eurotest was 77% (95% CI 68% to 85%) in people classed as not dementia, but only 36% (95% CI 18% to 58%) in people who GPs thought had dementia.

### Natural frequency classification

Supplementary Table 2 shows the natural frequency classification of people in a hypothetical population of 1000 people. Based on our data 550 of the 1000 people have dementia, GPs would classify 358 of the 1000 as having dementia, being correct in 308 of the 358. Without taking GP judgement into account SPMT is the test with best classification, leading to half (526) testing positive and potentially needing referral, of these 426 would be true positives (TP) with 100 false positives (FP); there would also be 351 true negatives (TN), and 125 false negatives (FN). Taking GP judgement into account then in the 358 people GPs classify as having dementia M@T is the test with best classification, whereas Eurotest has best classification in the 642 people classified as not having dementia. Combining M@T in the 358 classed by GPs as having dementia and Eurotest in 642 classed by GPs no dementia results in a total of 414 TP and 75 FP, with 376 TN and 138 FN.

Supplementary Table 3 gives the results of the exploratory bootstrapping procedure and indicates that for the tests under evaluation, there is a general trend that the stratified approach has fewer false classifications, though these differences could still be consistent with chance. For SPMT and CIT as the unstratified test, the reduction in false classifications is attributable to fewer false negatives, whereas for GPCOG there are fewer false positives with the stratified approach. With GPCOG (and to a lesser extend SPMT) as the unstratified comparison, the stratified approach has a trade-off between FP and FN whereas with CIT the trade-off is less clear, and stratification typically leads to fewer FP and FN.

Supplementary Table 4 presents a STARD checklist. Supplementary Table 6 presents a cross tabulation for each patient-index test against cognitive category.

## Discussion

This is the most comprehensive evaluation of a wide variety of tests for the diagnosis of dementia in people aged at least 70 years presenting to their GP with cognitive symptoms. We investigated the accuracy of eight BCAs, three physical tests and five informant measures, both alone and combined with GP judgement. Combining tests with GP judgement altered test accuracy.

There are several methodological strengths: patient selection is applicable to clinical practice, we verified all cases against the same reference standard, and there was adjudication of the reference standard for uncertain cases. We randomised the order of the index battery to minimise order effect. However, there are important limitations. We did not include people who were unable to attend with an informant, or people with severe cognitive impairment who were unable to consent, and so our findings cannot easily be generalised to them, especially regarding test duration and informant tests. There was no evidence of bias due to selective recruitment by GPs, or due to selective participation by cognitive status, but any systematic bias in recruitment would limit the generalisability of our findings to the people who were excluded [19]. We do not know whether GPs used any index tests prior to referral but based on previous studies, clinical judgement is likely to be based on rules of thumb [45], not formal tests [46], and information on referral forms indicated that judgement was informed by “face to face presentation”. There is insufficient power to detect statistically significant differences between test accuracy and the confidence intervals for tests overlap. Test accuracy may vary between generations, for example when using prime ministers, or currency, which are subject to change. A further important limitation is that despite providing translation services the population were largely white, native English speakers.

Most comparable studies have reported the accuracy of single tests. For example, MoCA at a threshold of 26 was reported to have a sensitivity of around 94% and specificity of at most 60%, but this was based on studies in secondary care which are likely to have a more severe spectrum of disease [36]. IQCODE at a threshold of 3.2 has been reported to have sensitivity 100% specificity 76% [47] and Eurotest at a threshold of 21 has been reported to have sensitivity 91% specificity 82% [20]. These results are comparable with ours. In a multi-test primary-care based study that included 47 people with dementia out of a total of 141 people, Eurotest took an average 7 minutes and Phototest an average 3 minutes in someone with dementia, and both tests had comparable accuracy [48], which compares well with our findings.

While we have only tested a few comparisons with our exploratory bootstrapping procedure, our data suggest that stratification by GP Judgement may help to reduce incorrect diagnostic classifications, but the interplay between judgement and the test will determine the impact. Although it is not possible to make firm recommendations about tests, we believe that BCAs such as M@T, Eurotest, and 6CIT and GPCOG may be particularly useful to consider for further investigation or use in practice; the last two tests may be particularly useful if time is highly valued in practice. These four tests have high predictive values in this setting, and our results suggest that these tests may be particularly useful when GP judgement is used to stratify people for further testing, but this requires confirmation in a future study.

One important implication is that there is substantial variation in duration of brief cognitive assessments when performed in this setting. Clinicians who are short of time may prefer to be familiar with the use of (and limitations of) a test which they can reliably do in less than 5 minutes in 95% of people, such as GPCOG, which when combined with clinical judgment has high specificity but only modest sensitivity. A second implication is that GP judgement could inform the selection of future tests because GP judgement has an important impact on prevalence and therefore predictive values (and diagnostic accuracy) of tests. The most time efficient diagnostic procedure while retaining high accuracy would be to stratify by GP judgement and use IQCODE in people who GPs judge as having dementia and 6CIT in people who GPs judge as having no dementia.

Further research in primary care to investigate the accuracy of tests for dementia in combination with GP judgement and each other is important to help refine our results and reduce the uncertainty in the estimates. Future research should attempt to identify the most discriminative tests to distinguish dementia and normal from MCI in people who in the clinical judgement of a GP have cognitive impairment but not having dementia, and also investigate the effect of GP stratification of particular tests on diagnostic accuracy, with particular attention given to the time taken to complete tests. We believe that it may be helpful to focus future research on tests such as M@T, Eurotest, 6CIT and GPCOG for reasons discussed above.

## Supplementary Material

Supplementary Table 1

Supplementary Table 2

Supplementary Table 3

Supplementary Table 4

Supplementary Table 6

## Figures and Tables

**Table 1:** Characteristics of participants by cognitive category by reference standard

	Dementia	CIND	Normal
	n= 132	n=61	n=47
*Sex n (column %)*			
Male	68 (51)	35 (57)	23 (49)
Female	64 (49)	26 (43)	24 (51)
*Age*			
At clinic (years) Median (IQR)	82 (77-86)	80 (75-83)	75 (72-80)
Age of leaving education (years) Median (IQR)	15 (15-16)	15 (15-16)	16 (15-16)
*Symptom onset Median (IQR) (months)*			
Time ago	24 (12-36)	18 (12-24)	21 (12-36)
*ACE-III Score median (IQR)*			
Total (max 100)	69 (61-74)	82 (76-87)	93 (90-95)
*GP Judgement n (row %)*			
Normal	6 (18)	9 (26)	19 (56)
CIND	52 (43)	41 (34)	27 (23)
Dementia	74 (86)	11 (13)	1(1)

Dementia according to ICD-10 [40] ACE-III Addenbrooke’s’ Cognitive Examination III; CIND Cognitive impairment, not dementia.

**Table 2:** Accuracy of brief cognitive tests for the diagnosis of dementia

	Alone		With GP Judgement*	
	Sensitivity (95% CI)	Specificity (95% CI)	Sensitivity (95% CI)	Specificity (95% CI)
Cognitive or physical tests				
*Brief tests, 95%. Completed within less than 5 minutes*				
EPSS	85 (78 to 90)	30 (21 to 39)	44 (35 to 53)	91 (84 to 95)
T&C	27 (20 to 36)	96 (91 to 99)	17 (11 to 24)	100 (97 to 100)
Phototest	57 (48 to 66)	82 (74 to 89)	37 (29 to 46)	94 (88 to 98)
6CIT	76 (67 to 83)	70 (60 to 79)	46 (37 to 55)	93 (86 to 97)
GPCOG	93 (87 to 97)	52 (42 to 62)	55 (46 to 64)	91 (84 to 95)
Mini-cog	70 (61 to 77)	73 (64 to 81)	42 (34 to 51)	94 (87 to 97)
*Medium tests, 95% completed within ≥5 but ≤10 minutes*				
Eurotest	70 (62 to 78)	81 (72 to 88)	44 (35 to 53)	97 (92 to 99)
SPMT	77 (69 to 84)	78 (69 to 85)	49 (40 to 58)	96 (91 to 99)
TUG	23 (16 to 31)	91 (84 to 95)	14 (9 to 21)	98 (93 to 100)
Sniffin’ Sticks	100 (96 to 100)	4 (1 to 11)	51 (41 to 61)	88 (80 to 94)
*Longer tests 95% completed within more than 10 minutes*				
M@T	63 (41 to 81)	80 (44 to 97)	54 (33 to 74)	100 (69 to 100)
MoCA	100 (97 to 100)	16 (10 to 25)	55 (45 to 64)	87 (77 to 93)
GP Judgement	56 (47 to 65)	89 (81 to 94)	-	-
Informant data				
FAQ	27 (19 to 35)	97 (92 to 99)	19 (13 to 27)	98 (93 to 100)
Katz ADL	26 (19 to 34)	95 (90 to 98)	15 (9 to 22)	99 (95 to 100)
Lawton IADL	37 (29 to 46)	87 (79 to 93)	26 (19 to 34)	96 (91 to 99)
AD8	96 (91 to 99)	32 (24 to 42)	55 (46 to 63)	92 (85 to 96)
IQCODE	95 (90 to 98)	38 (28 to 48)	56 (47 to 64)	91 (84 to 96)

* Combined test positive was taken as being both GP judgement positive and the other test positive; and the combined test negative was taken as either both or one of judgement/test negative.

See Supplementary Table 1 for test abbreviations. CI confidence interval

**Table 3:** Discrimination of tests, by GP judgement

TEST	AREA UNDER ROC CURVE (AUROC) (95% confidence interval) Results 10^-2^			PPV FOR DEMENTIA (95% confidence interval) Results 10^-2^		NPV FOR DEMENTIA (95% confidence interval) Results 10^-2^	
	Overall (n=240)	GP dementia (n=86)	GP not dementia (n=154)	GP dementia (n=86)	GP not dementia (n=154)	GP dementia (n=86)	GP not dementia (n=154)
**EPSS**	57 (52 to 63)	48 (36 to 60)	62 (56 to 68)	85 (75 to 93)	45 (36 to 54)	11 (1 to 35)	88 (73 to 97)
**TAC**	62 (58 to 66)	65 (60 to 70)	60 (54 to 66)	100 (85 to 100)	78 (52 to 94)	19 (10 to 31)	68 (59 to 75)
**6CIT**	73 (67 to 79)	58 (43 to 72)	71 (63 to 78)	88 (78 to 95)	62 (49 to 74)	24 (7 to 50)	79 (69 to 87)
**GPCOG**	73 (67 to 78)	58 (47 to 69)	71 (65 to 78)	88 (79 to 94)	54 (44 to 65)	67 (9 to 99)	87 (76 to 94)
**SPMT**	78 (72 to 83)	77 (63 to 92)	71 (64 to 79)	94 (86 to 98)	65 (51 to 77)	47 (23 to 72)	78 (69 to 86)
**TUG**	57 (52 to 61)	54 (42 to 66)	56 (50 to 62)	90 (68 to 99)	58 (34 to 80)	15 (8 to 27)	67 (58 to 75)
**M@T**	71 (53 to 88)	93 (. to 100)	50 (30 to 70)	100 (75 to 100)	50 (7 to 93	33 (1 to 91)	50 (23 to 77)
**MOCA**	58 (55 to 62)	. (. to .)	59 (55 to 63)	. (. to .)	41 (32 to 50)	. (. to .)	100 (79 to 100)
**PHOTOTEST**	70 (64 to 75)	58 (42 to 74)	66 (59 to 73)	89 (78 to 96)	67 (50 to 81)	19 (7 to 36)	73 (63 to 81)
**MINICOG**	71 (66 to 77)	59 (43 to 74)	70 (62 to 77)	89 (78 to 95)	62 (48 to 75)	22 (7 to 44)	77 (67 to 85)
**EUROTEST**	76 (71 to 81)	77 (63 to 90)	71 (63 to 78)	95 (86 to 99)	66 (52 to 79)	36 (18 to 58)	77 (68 to 85)
**SNIFFIN’**	52 (50 to 54)	. (. to .)	53 (50 to 55)	. (. to .)	39 (30 to 48)	. (. to .)	100 (40 to 100)
**FAQ**	62 (58 to 66)	59 (46 to 71)	58 (53 to 63)	93 (76 to 99)	91 (59 to 100)	17 (8 to 29)	66 (58 to 74)
**ADL**	61 (56 to 65)	59 (49 to 68)	61 (55 to 67)	95 (75 to 100)	79 (54 to 94)	17 (9 to 28)	69 (60 to 76)
**IADL**	62 (57 to 67)	56 (41 to 71)	58 (51 to 64)	90 (75 to 97)	60 (39 to 79)	17 (7 to 30)	67 (58 to 75)
**AD8**	64 (60 to 69)	61 (48 to 74)	64 (59 to 70)	89 (80 to 95)	46 (37 to 56)	60 (15 to 95)	91 (77 to 98)
**IQCODE**	67 (62 to 72)	55 (45 to 65)	65 (59 to 72)	89 (80 to 95)	50 (40 to 60)	100 (3 to 100)	86 (72 to 95)

PPV positive predictive value NPV negative predictive value See Supplementary Table 1 for test abbreviations. Missing data (.) are where the value is not computable. e.g., for example no cases where GPs judged a diagnosis of dementia were test-normal on MoCA

## Data Availability

The datasets generated and analysed during the current study are not yet publicly available as the funder approved pre-specified data management plan stated we would embargo access for five years, but are available from the corresponding author on reasonable request. Data will be available from the data.Bris repository after an embargo period of five years. Statistical code is available on request from the authors.
