# Supplementary material for: A diagnostic test accuracy study investigating GP clinical impression and brief cognitive assessments for dementia in primary care, compared to specialised assessment
Source: J Alzheimers Dis. Author manuscript; Available in PMC 2023 Nov 7. (PMC7615275; doi:10.3233/JAD-230320)
Supplement: Supplementary Table 1 [file EMS184937-supplement-Supplementary_Table_1.docx]

**Supplementary Table 1. Characteristics of tests by cognitive category assigned by reference standard**

|  | Cognitive category | | |
| --- | --- | --- | --- |
|  | Dementia  N=61 | CIND  N=47 | *Normal*  *N=132* |
|  | Difference, compared to normal | | *x*¯ (sd) *‡* |
| **M@T *n=34* [0-50] *⇑τ* <28** | | | |
| Test score (95% CI) | -13 (-20 to -5.4) | -7.0 (-16 to 1.8) | 39 (3.7) |
| Test duration (95% CI) | +3.2 (1.4 to 5.1) | +1.9 (-0.29 to 4.1) | 4.8 (0.50) |
| **MOCA *n=206* [0-30] *⇑τ* <26** | | | |
| Test score (95% CI) | -8.7 (-10 to -7.2) | -3.8 (-5.6 to -2.1) | 24 (2.7) |
| Test duration (95% CI) | +2.6 (1.8 to 3.3) | +1.3 (0.50 to 2.2) | 9.4 (1.4) |
| **Eurotest *n=240* [0-35] *⇑τ* <21** | | | |
| Test score (95% CI) | -11 (-13 to -9.0) | -4.5 (-6.7 to -2.4) | 27 (3.5) |
| Test duration (95% CI) | +1.9 (1.5 to 2.4) | +1.1 (0.56 to 1.7) | 5.6 (1.2) |
| **Time & Change *n=240 ⇑τ* <2** | | | |
| Pass (n, col %) | 95 (73) | 55 (93) | 47 (100) |
| Test duration (95% CI) | +0.55 (0.31 to 0.80) | +0.28 (-0.01 to 0.56) | 1.5 (0.55) |
| **Phototest *n=238* [0-*∞*] *⇑τ* <27** | | | |
| Test score (95% CI) | -12 (-15 to -10) | -6.0 (-8.7 to -3.3) | 37 (6.2) |
| Test duration (95% CI) | +0.22 (0.008 to 0.43) | +0.24 (0.005 to 0.48) | 2.8 (0.54) |
| **SPMT *n=240* [0-23] *⇑τ* <10** | | | |
| Test score (95% CI) | -7.9 (-9.2 to -6.6) | -3.8 (-5.2 to -2.3) | 15 (3.5) |
| Test duration (95% CI) | +0.03 (-0.46 to 0.47) | +0.02 (-0.52 to 0.56) | 6.6 (1.2) |
| **CIT *n=238* [0-28] *⇓τ* >7** | | | |
| Test score (95% CI) | 9.8 (8.0 to 12) | 5.1 (3.0 to 7.3) | 2.6 (2.9) |
| Test duration (95% CI) | +0.51 (0.28 to 0.75) | +0.25 (-0.02 to 0.52) | 1.1 (0.64) |
| **Minicog *n=240* [0-5] *⇑τ* <3** | | | |
| Test score (95% CI) | -2.5 (-3.0 to -2.1) | -1.4 (-2.0 to -0.88) | 4.5 (0.8) |
| *Duration not available as calculated* | | | |
| **GPCOG *n=240* [0-9] *⇑τ ∗∗*** | | | |
| Test score (95% CI) | -4.4 (-5.1 to -3.7) | -2.4 (-3.2 to -1.5) | 8.1 (1.2) |
| Test duration (95% CI) | +1.1 (0.80 to 1.5) | +0.66 (0.28 to 1.0) | 1.8 (0.60) |
| **TUG *n=236* [0-*∞*] *⇓τ* > 15** | | | |
| Test time, seconds (95% CI) | 4.6 (2.6 to 6.7) | 2.7 (0.3 to 5.0) | 8.8 (2.1) |
| Test duration (95% CI) | +0.34 (-0.02 to 0.70) | +0.15 (-0.27 to 0.56) | 2.4 (1.1) |
| **EPSS *n=240* [0-28] *⇓τ* > 1** | | | |
| Test score (95% CI) | 3.2 (2.1 to 4.3) | 2.0 (0.78 to 3.3) | 2.3 (1.8) |
| Test duration (95% CI) | +0.18 (-0.05 to 0.41) | -0.06 (-0.33 to 0.20) | 1.0 (0.53) |
| **Sniffin sticks *n=188* [0-16] *⇑τ* < 11** | | | |
| Test score (95% CI) | -2.2 (-3.1 to 1.3) | -1.6 (-2.6 to -0.59) | 7.9 (2.6) |
| Test duration (95% CI) | +0.42 (-0.05 to 0.90) | +0.65 (0.11 to 1.2) | 4.0 (1.0) |

*∗* as assigned by expert judgement according to ICD-10 criteria for dementia.

CIND Cognitive Impairment Not Dementia, most of whom had MCI (Mild cognitive impairment)

*†* For test name abbreviations see text. [x-y] score range. Test duration (minutes) better cognition indicated by *⇓* lower scores and *⇑* higher scores.

*τ* threshold score for abnormal

** for GPCOG a two stage approach to scoring is used whereby scores >8 indicate normal and <5 indicate abnormal and scores 5-8 indicate GPCOGi needed, where scores <4 indicate abnormal

Duration for Mini-cog not given because the score was calculated using items from the GPCOG
