# Supplementary material for: A diagnostic test accuracy study investigating GP clinical impression and brief cognitive assessments for dementia in primary care, compared to specialised assessment
Source: J Alzheimers Dis. Author manuscript; Available in PMC 2023 Nov 7. (PMC7615275; doi:10.3233/JAD-230320)
Supplement: Supplementary Table 2 [file EMS184937-supplement-Supplementary_Table_2.docx]

Supplementary Table 2: natural frequency classification of people in a population of approximately 1000 people presenting with symptoms of possible dementia

|  | TP | TN | FP | FN |
| --- | --- | --- | --- | --- |
| *Regardless of time* | | | | |
| No GP judgement (n=1000) |  |  |  |  |
| SPMT | 426 | 351 | 100 | 125 |
| Eurotest | 388 | 363 | 88 | 163 |
| GP = dementia (n=358) |  |  |  |  |
| [M@T](mailto:M@T) | 268 | 50 | 0 | 42 |
| GPCOG | 305 | 9 | 42 | 5 |
| GP = not dementia (n=642) |  |  |  |  |
| Eurotest | 146 | 326 | 75 | 96 |
| SPMT | 155 | 317 | 84 | 88 |
| *Accounting for time* | | | | |
| No GP judgement (n=1125 assessed) |  |  |  |  |
| IQCODE | 591 | 193 | 314 | 29 |
| AD8 | 596 | 165 | 343 | 24 |
| 6CIT | 312 | 237 | 101 | 101 |
| GP = dementia (n= 1125 assessed) |  |  |  |  |
| IQCODE | 969 | 16 | 142 | 0 |
| AD8 | 942 | 40 | 118 | 27 |
| 6CIT | 340 | 113 | 226 | 74 |
| GP = not dementia (n= 1125 assessed) |  |  |  |  |
| 6CIT | 416 | 379 | 129 | 203 |
| ADL | 112 | 672 | 30 | 313 |
| TAC | 100 | 324 | 15 | 314 |

*Numbers may not sum due to rounding TP True positives TN True negatives FP False Positives FN False negatives See Supplementary Table 1 for test abbreviations*
