# Supplementary material for: A diagnostic test accuracy study investigating GP clinical impression and brief cognitive assessments for dementia in primary care, compared to specialised assessment
Source: J Alzheimers Dis. Author manuscript; Available in PMC 2023 Nov 7. (PMC7615275; doi:10.3233/JAD-230320)
Supplement: Supplementary Table 3 [file EMS184937-supplement-Supplementary_Table_3.docx]

**Supplementary Table 3**

**Bootstrapped comparisons of mis-classifications using tests unstratified, and stratified by GP judgement**

| **Unstratified ~ Stratified GP dementia \| Stratified GP normal** | **n in analysis** | **n normal** | **n dementia** | **n FP + FN (95% CI) p for comparison** | **Difference in FP (unstratified - stratified) p for comparison** | **Difference in FN (unstratified - stratified)** |
| --- | --- | --- | --- | --- | --- | --- |
| spmt ~ gpcog \| eurotest | 240 | 108 | 132 | 2 (-12.21, 16.21); p=0.78 | -4 (-14.69, 6.69); p=0.46 | 6 (-3.38, 15.38); p=0.21 |
| spmt ~ gpcog \| cit | 238 | 107 | 131 | 0 (-14.69, 14.69); p=1.00 | -10 (-20.27, 0.27); p=0.056 | 10 (-0.50, 20.50); p=0.062 |
| spmt ~ gpcog \| spmt | 240 | 108 | 132 | 2 (-5.77, 9.77); p=0.61 | -6 (-10.90, -1.10); p=0.016 | 8 (2.39, 13.61); p=0.005 |
| spmt ~ ad8 \| eurotest | 240 | 108 | 132 | 2 (-13.68, 17.68); p=0.80 | -3 (-14.38, 8.38); p=0.61 | 5 (-5.28, 15.28); p=0.34 |
| spmt ~ ad8 \| cit | 238 | 107 | 131 | 0 (-16.15, 16.15); p=1.00 | -9 (-20.50, 2.50); p=0.12 | 9 (-2.18, 20.18); p=0.11 |
| spmt ~ ad8 \| spmt | 240 | 108 | 132 | 2 (-6.86, 10.86); p=0.66 | -5 (-10.97, 0.97); p=0.10 | 7 (0.53, 13.47); p=0.034 |
| spmt ~ iqcode \| eurotest | 231 | 100 | 131 | 3 (-11.50, 17.50); p=0.69 | -4 (-14.11, 6.11); p=0.44 | 7 (-2.81, 16.81); p=0.16 |
| spmt ~ iqcode \| cit | 229 | 99 | 130 | 0 (-14.95, 14.95); p=1.00 | -11 (-21.13, -0.87); p=0.033 | 11 (0.48, 21.52); p=0.040 |
| spmt ~ iqcode \| spmt | 231 | 100 | 131 | 2 (-5.77, 9.77); p=0.61 | -7 (-11.97, -2.03); p=0.006 | 9 (3.30, 14.70); p=0.002 |
| cit ~ gpcog \| eurotest | 238 | 107 | 131 | 12 (-2.90, 26.90); p=0.11 | 4 (-5.78, 13.78); p=0.42 | 8 (-2.67, 18.67); p=0.14 |
| cit ~ gpcog \| cit | 238 | 107 | 131 | 10 (2.14, 17.86); p=0.013 | -2 (-5.97, 1.97); p=0.32 | 12 (5.34, 18.66); p<0.001 |
| cit ~ gpcog \| spmt | 238 | 107 | 131 | 12 (-2.64, 26.64); p=0.11 | 2 (-7.83, 11.83); p=0.69 | 10 (-1.01, 21.01); p=0.075 |
| cit ~ ad8 \| eurotest | 238 | 107 | 131 | 12 (-3.72, 27.72); p=0.13 | 5 (-5.30, 15.30); p=0.34 | 7 (-4.49, 18.49); p=0.23 |
| cit ~ ad8 \| cit | 238 | 107 | 131 | 10 (1.27, 18.73); p=0.025 | -1 (-5.54, 3.54); p=0.67 | 11 (3.49, 18.51); p=0.004 |
| cit ~ ad8 \| spmt | 238 | 107 | 131 | 12 (-3.62, 27.62); p=0.13 | 3 (-7.25, 13.25); p=0.57 | 9 (-2.87, 20.87); p=0.14 |
| cit ~ iqcode \| eurotest | 229 | 99 | 130 | 13 (-0.63, 26.63); p=0.062 | 4 (-5.12, 13.12); p=0.39 | 9 (-1.57, 19.57); p=0.095 |
| cit ~ iqcode \| cit | 229 | 99 | 130 | 10 (2.37, 17.63); p=0.010 | -3 (-6.35, 0.35); p=0.079 | 13 (6.35, 19.65); p<0.001 |
| cit ~ iqcode \| spmt | 229 | 99 | 130 | 12 (-2.03, 26.03); p=0.094 | 1 (-7.98, 9.98); p=0.83 | 11 (-0.25, 22.25); p=0.055 |
| gpcog ~ gpcog \| eurotest | 240 | 108 | 132 | 9 (-5.16, 23.16); p=0.21 | 24 (13.24, 34.76); p<0.001 | -15 (-22.90, -7.10); p<0.001 |
| gpcog ~ gpcog \| cit | 238 | 107 | 131 | 7 (-5.04, 19.04); p=0.25 | 18 (8.39, 27.61); p<0.001 | -11 (-18.16, -3.84); p=0.003 |
| gpcog ~ gpcog \| spmt | 240 | 108 | 132 | 9 (-4.72, 22.72); p=0.20 | 22 (10.97, 33.03); p<0.001 | -13 (-20.47, -5.53); p<0.001 |
| gpcog ~ ad8 \| eurotest | 240 | 108 | 132 | 9 (-5.64, 23.64); p=0.23 | 25 (13.83, 36.17); p<0.001 | -16 (-24.71, -7.29); p<0.001 |
| gpcog ~ ad8 \| cit | 238 | 107 | 131 | 7 (-6.32, 20.32); p=0.30 | 19 (8.74, 29.26); p<0.001 | -12 (-20.05, -3.95); p=0.003 |
| gpcog ~ ad8 \| spmt | 240 | 108 | 132 | 9 (-5.23, 23.23); p=0.22 | 23 (11.93, 34.07); p<0.001 | -14 (-22.60, -5.40); p=0.001 |
| gpcog ~ iqcode \| eurotest | 231 | 100 | 131 | 9 (-4.64, 22.64); p=0.20 | 23 (12.73, 33.27); p<0.001 | -14 (-21.98, -6.02); p<0.001 |
| gpcog ~ iqcode \| cit | 229 | 99 | 130 | 6 (-6.05, 18.05); p=0.33 | 16 (6.57, 25.43); p<0.001 | -10 (-17.27, -2.73); p=0.007 |
| gpcog ~ iqcode \| spmt | 231 | 100 | 131 | 8 (-6.04, 22.04); p=0.26 | 20 (9.30, 30.70); p<0.001 | -12 (-20.39, -3.61); p=0.005 |
|  |  |  |  |  |  |  |
| A positive number means that the unstratified approach has a larger number of that variable. So if the number is 4 in column FP that means the number of false-positives was four higher in the unstratified approach (or you could say stratified approach reduces the number of mis-classifications) | | | | | | |
