# Supplementary material for: A diagnostic test accuracy study investigating GP clinical impression and brief cognitive assessments for dementia in primary care, compared to specialised assessment
Source: J Alzheimers Dis. Author manuscript; Available in PMC 2023 Nov 7. (PMC7615275; doi:10.3233/JAD-230320)
Supplement: Supplementary Table 4 [file EMS184937-supplement-Supplementary_Table_4.docx]

**Supplementary Table 5: STARD checklist**

| **Section, topic, and item number** | **STARD checklist item** | **Page** |
| --- | --- | --- |
| 1 | Identify the article as a study of diagnostic accuracy (recommend MeSH heading “ sensitivity and specificity”) | 1 |
| 2 | State the research questions or study aims, such as estimating diagnostic accuracy or comparing accuracy between tests or across participant groups | 1 |
| 3 | The study population: the inclusion and exclusion criteria, setting and locations where data were collected. See also item 4 on recruitment and item 5 on sampling | 1 |
| 4 | Participant recruitment: Was recruitment based on presenting symptoms, results from previous tests, or the fact that the participants had received the index tests or the reference standard? See also item 5 on sampling and item 16 on participant loss at each stage of the study | 2 |
| 5 | Participant sampling: Was the study population a consecutive series of participants defined by the selection criteria in items 3 and 4? If not, specify how participants were further selected. See also item 4 on recruitment and item 16 on participant loss | 2 |
| 6 | Data collection: Was data collection planned before the index test and reference standard were performed (prospective study) or after (retrospective study)? | 2 |
| 7 | The reference standard and its rationale | 2 |
| 8 | Technical specifications of materials and methods involved including how and when measurements were taken, and/or cite references for index tests and reference standard. See also item 10 concerning the person(s) executing the tests | 2 |
| 9 | Definition of and rationale for the units, cutoffs, and/or categories of the results of the index tests and the reference standard | 2 |
| 10 | The number, training, and expertise of the persons executing and reading the index tests and the reference standard. See also item 8 | 2 |
| 11 | Whether or not the readers of the index tests and reference standard were blinded (masked) to the results of the other test and describe any other clinical information available to the readers. See also item 7 | 2 |
| 12 | Methods for calculating or comparing measures of diagnostic accuracy, and the statistical methods used to quantify uncertainty (e.g., 95% confidence intervals) | 2 |
| 13 | Methods for calculating test reproducibility, if done | NA |
| 14 | When study was performed, including beginning and end dates of recruitment | 3 |
| 15 | Clinical and demographic characteristics of the study population (at least information on age, sex, spectrum of presenting symptoms). See also item 18 | 5 |
| 16 | The number of participants satisfying the criteria for inclusion who did or did not undergo the index tests and/or the reference standard; describe why participants failed to undergo either test (a flow diagram is strongly recommended). See also items 3–5 | 6 |
| 17 | Time interval between the index tests and the reference standard, and any treatment administered in between | 3 |
| 18 | Distribution of severity of disease (define criteria) in those with the target condition; other diagnoses in participants without the target condition | 6 |
| 19 | A cross-tabulation of the results of the index tests (including indeterminate and missing results) by the results of the reference standard; for continuous results, the distribution of the test results by the results of the reference standard | 6 |
| 20 | Any adverse events from performing the index tests or the reference standard | 3 |
| 21 | Estimates of diagnostic accuracy and measures of statistical uncertainty (e.g., 95% confidence intervals). See also item 12 | 9 |
| 22 | How indeterminate results, missing data, and outliers of the index tests were handled | 3 |
| 23 | Estimates of variability of diagnostic accuracy between subgroups of participants, readers, or centers, if done | 8 |
| 24 | Estimates of test reproducibility, if done. See also item 13 | NA |
| 25 | Discuss the clinical applicability of the study findings | 3-4 |
