# Supplementary material for: A diagnostic test accuracy study investigating GP clinical impression and brief cognitive assessments for dementia in primary care, compared to specialised assessment
Source: J Alzheimers Dis. Author manuscript; Available in PMC 2023 Nov 7. (PMC7615275; doi:10.3233/JAD-230320)
Supplement: Supplementary Table 6 [file EMS184937-supplement-Supplementary_Table_6.docx]

**Supplementary Table 6. Cross tab for each index test by cognitive category assigned by reference standard**

|  | Cognitive category | | | | | |
| --- | --- | --- | --- | --- | --- | --- |
|  | Dementia  N=61 | | CIND  N=47 | | *Normal*  *N=132* | |
|  | Difference, compared to normal | | | | *x*¯ (sd) *‡* | |
| **M@T *n=34* [0-50] *⇑τ* <28** | | | | | | |
| Test positive | 15 | | 2 | | 0 | |
| Test negative | 9 | | 4 | | 4 | |
| **MOCA *n=206* [0-30] *⇑τ* <26** | | | | | | |
| Test positive | 108 | | 50 | | 32 | |
| Test negative | 0 | | 5 | | 11 | |
| **Eurotest *n=240* [0-35] *⇑τ* <21** | | | | | | |
| Test positive | 93 | | 19 | | 2 | |
| Test negative | 39 | | 42 | | 45 | |
| **Time & Change *n=240 ⇑τ* <2** | | | | | | |
| Test positive | 36 | | 4 | | 0 | |
| Test negative | 96 | | 57 | | 47 | |
| **Phototest *n=238* [0-*∞*] *⇑τ* <27** | | | | | | |
| Test positive | 75 | | 15 | | 4 | |
| Test negative | 56 | | 46 | | 42 | |
| **SPMT *n=240* [0-23] *⇑τ* <10** | | | | | | |
| Test positive | 102 | | 22 | | 2 | |
| Test negative | 30 | | 39 | | 45 | |
| **CIT *n=238* [0-28] *⇓τ* >7** | | | | | | |
| Test positive | 99 | | 30 | | 2 | |
| Test negative | 32 | | 31 | | 44 | |
| **Minicog *n=240* [0-5] *⇑τ* <3** | | | | | | |
| Test positive | | 92 | | 27 | | 2 |
| Test negative | | 40 | | 34 | | 45 |
| **GPCOG *n=240* [0-9] *⇑τ ∗∗*** | | | | | | |
| Test positive | 123 | | 43 | | 9 | |
| Test negative | 9 | | 18 | | 38 | |
| **TUG *n=236* [0-*∞*] *⇓τ* > 15** | | | | | | |
| Test positive | 29 | | 9 | | 1 | |
| Test negative | 99 | | 52 | | 46 | |
| **EPSS *n=240* [0-28] *⇓τ* > 1** | | | | | | |
| Test positive | 112 | | 49 | | 27 | |
| Test negative | 20 | | 12 | | 20 | |
| **Sniffin sticks *n=188* [0-16] *⇑τ* < 11** | | | | | | |
| Test positive | 98 | | 48 | | 38 | |
| Test negative | 0 | | 1 | | 3 | |

*∗* as assigned by expert judgement according to ICD-10 criteria for dementia.

CIND Cognitive Impairment Not Dementia, most of whom had MCI (Mild cognitive impairment)

*†* For test name abbreviations see text. [x-y] score range. Test duration (minutes) better cognition indicated by *⇓* lower scores and *⇑* higher scores.

*τ* threshold score for abnormal

** for GPCOG a two stage approach to scoring is used whereby scores >8 indicate normal and <5 indicate abnormal and scores 5-8 indicate GPCOGi needed, where scores <4 indicate abnormal

Duration for Mini-cog not given because the score was calculated using items from the GPCOG
